# Commentary on “Reflections on the Inclusion of Direct-Care Physicians as Educators in Community Hospitals”

**DOI:** 10.1590/0100-6991e-20243865-en

**Published:** 2024-12-02

**Authors:** ROSANA ALVES

**Affiliations:** 1 - Centro Universitário FAESA - Vitória - ES - Brasil.; 2 - Instituto Capixaba de Ensino, Pesquisa e Inovação - Vitória - ES - Brasil.

**Keywords:** Education, Medical, Preceptorship, Hospitals, Community, Unified Health System, Schools, Medical, Educação Médica, Preceptoria, Hospitais Comunitários, Sistema Único de Saúde, Faculdades de Medicina

## Abstract

The article by Sweigart et al. presents concerns, challenges, and proposals for the current situation, both nationally and internationally, and the need for a diversity of medical care practice scenarios that simultaneously develop teaching abilities. Medical education is now conducted outside the university-affiliated teaching hospital, often in the so-called community or general hospitals dedicated to patient care. In Brazil, most of these hospitals are under municipal or state management.

## RESUMO

O artigo de Sweigart et al. apresenta preocupações, desafios e proposições para o momento em que nos encontramos, tanto no cenário nacional como internacional, a necessidade de uma diversidade de cenários de prática médica assistenciais que desenvolvam concomitantemente, o ensino. A formação médica passa a ocorrer fora do hospital vinculado à escola médica, em hospitais vocacionados para a assistência, chamados no artigo como comunitários ou gerais. No Brasil, em sua maioria, estes hospitais estão sob gestão municipal ou estadual.

## INTRODUCTION

The challenge lies in transforming healthcare facilities into teaching-care environments, requiring physicians in general hospitals-those responsible for daily inpatient care, emergency departments, family health teams, or even specialty reference centers-to balance clinical practice with teaching responsibilities. Physicians who were previously focused solely on patient care are now taking on teaching roles, often reluctantly or without prior inclination, as a result of the expansion of medical schools and residency programs.

In Brazil, following the publication of the Brazilian National Curricular Guidelines for undergraduate medical education (Brazil, 2001; Brazil, 2014), medical training began to encompass all three levels of care, emphasizing internships supervised by practicing physicians, known as preceptors. Additionally, the number of medical schools increased due to a combination of factors, including public health demands, government policies, economic incentives, and the broader expansion of higher education in the country. Key drivers included the need to meet the growing demand for healthcare within the Brazilian Unified Health System (SUS), particularly in underserved areas such as the North and Northeast; the “Mais Médicos” program, launched in 2013 to address physician shortages in various parts of the country, which encouraged the opening of new medical schools, particularly in the private sector; where institutions saw an opportunity for high profitability of medical courses due to intense demand and steep tuition fees. Currently, Brazil has 374 undergraduate medical programs (Brazil, 2013; Roberti, 2016).

While this expansion has improved access to medical education, it has also introduced challenges such as maintaining teaching quality and ensuring adequate supervision in clinical settings.

Physicians in these settings often struggle to balance the demands of teaching with patient care, requiring additional time and the development of pedagogical skills. The need for pedagogical training for preceptors is urgent, as is the development of accessible educational tools, such as instructional videos and brief lectures, to support learning in high-pressure clinical environments with limited opportunities for in-depth discussions. 

Another concerning issue is the lack of experience among newly graduated physicians. In Brazil, medical school graduates can immediately register with the Regional Medical Council and work in roles that do not require postgraduate training. These are newly graduated doctors, barely out of adolescence, entrusted with the lives of others (Rego, 2012), and authorized to provide care in Basic Health Units (UBS), Emergency Care Units (UPA), and even hospital wards, often without undergoing a selection process or having an established employment relationship. The relationship has become precarious and outsourced under the management of Social Organizations (OS), private law, non-profit companies (Brazil, 1998).

To compensate for the use of municipal or state healthcare facilities as training grounds, medical schools are expected to provide pedagogical training for service preceptors and technical support, such as updates to clinical protocols. 

The strategies for quality medical education for students and residents, in all practice settings, are described below:

- Clear learning objectives and competencies must be defined for both undergraduate and residency programs, with specific goals aligned to the curricula and a shared responsibility for public health outcomes. These objectives and competencies should be consistently communicated to health unit managers, as misalignment between teaching and care practices remains a persistent issue (Brazil, 2014; Brazil, 2021);
- Establishing structured training programs is crucial, where medical schools assume responsibility for the territories or municipalities they serve. These programs should align with scientific societies to ensure technical updates for preceptors, residents, and interns, following the National Policy for Continuing Education, which supports professionals in addressing the population’s health needs (Brazil, 2013a);
- Preceptor training should include opportunities for pedagogical development, supported by specific tools designed to facilitate teaching, as part of a national policy;
- Expectations in accreditation: Create more detailed standards for certification and/or accreditation requirements for healthcare units that provide education at the three levels of care, ensuring that the structure and professionals are adequately prepared for teaching.

## CONCLUSION

To effectively address ongoing challenges, continuous monitoring and alignment with planned solutions and strategies are essential, with regular supervision, quality indicator analysis, and feedback at practice sites to identify and address shortcomings. Supervision may be conducted internally by medical school faculty and residency program supervisors or externally by INEP/MEC and CNRM. However, Brazil needs to create a National Policy for the evaluation and monitoring of medical education, coordinated with relevant education and health organizations to enable consistent and immediate action.
